# The Pathophysiology of Low Systemic Blood Flow in the Preterm Infant

**DOI:** 10.3389/fped.2018.00029

**Published:** 2018-02-16

**Authors:** Martin Kluckow

**Affiliations:** ^1^Department of Neonatology, Royal North Shore Hospital, University of Sydney, Sydney, NSW, Australia

**Keywords:** preterm, neonate, hypotension, hemodynamics, superior vena cava

## Abstract

Assessment and treatment of the VLBW infant with cardiovascular impairment requires understanding of the underlying physiology of the infant in transition. The situation is dynamic with changes occurring in systemic blood pressure, pulmonary pressures, myocardial function, and ductal shunt in the first postnatal days. New insights into the role of umbilical cord clamping in the transitional circulation have been provided by large clinical trials of early versus later cord clamping and a series of basic science reports describing the physiology in an animal model. Ultrasound assessment is invaluable in assessment of the physiology of the transition and can provide information about the size and shunt direction of the ductus arteriosus, the function of the myocardium and its filling as well as measurements of the cardiac output and an estimate of the state of peripheral vascular resistance. This information not only allows more specific treatment but it will often reduce the need for treatment.

## Introduction

**Figure 1:**
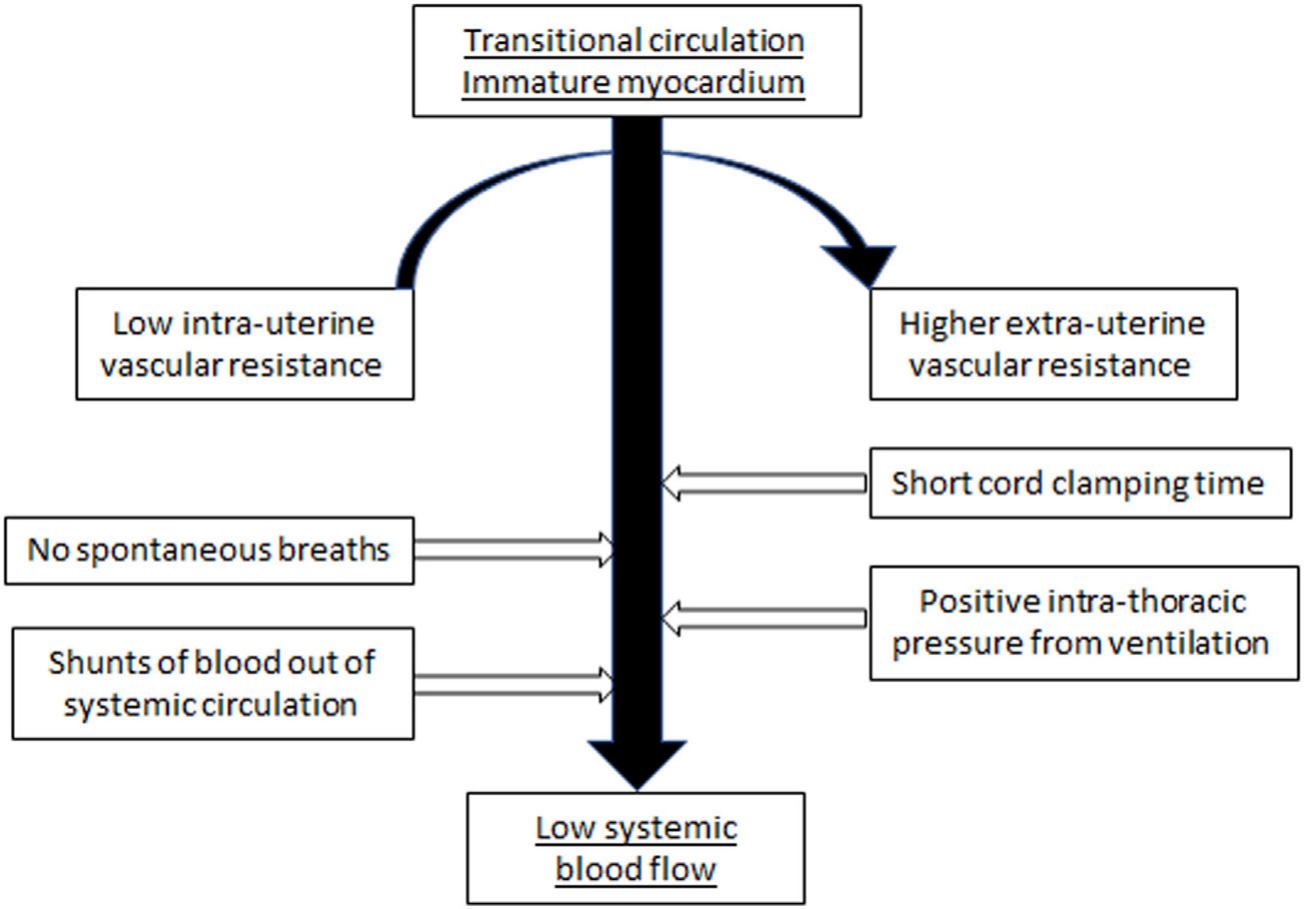
Suggested model of how the various external and internal influences on the cardiovascular system of the VLBW infant can result in low systemic blood flow.

The cardiovascular system of the fetus is adapted to an *in utero* environment that is constant and stable with the low resistance placenta being a key part of the hemodynamic equilibrium. The determinants of cardiac output are maintained in equilibrium without interference from external factors. Postnatal factors that can affect the cardiovascular function of the VLBW infant include perinatal asphyxia, sepsis, positive pressure respiratory support, variable carbon dioxide levels, and the time of clamping of the umbilical cord. During the immediate transitional period, these factors may alter preload, and change afterload at a time of rapid transition from the fetal circulation, characterized by low systemic vascular resistance, to the postnatal neonatal circulation with higher peripheral vascular resistance. The predominantly systemic to pulmonary shunts, occurring at atrial and ductal level through persisting fetal channels, can further reduce potential systemic blood flow (SBF) (Figure 1).

## Definition of Hypotension

Hypotension can be defined in two ways—physiologically or population based. The physiological definition is the blood pressure value where autoregulation of vital organ (brain) blood flow is reduced or lost with an impairment of adequate delivery of oxygen to the organs and tissues. If the blood pressure further decreases (and no treatment is initiated) it will eventually reach a “functional threshold” when neuronal function is impaired, and then finally an “ischemic threshold” which may result in tissue injury with likely permanent organ damage (1). From a clinical view point, neither of these limits have been well defined in the premature infant in the immediate postnatal period (2).

Low blood pressure may also be defined as population based. One definition is a mean blood pressure (MBP) less than 30 mm Hg in any gestation infant in the first postnatal days. This definition relates to previously observed pathophysiologic associations between cerebral injury (white matter damage, periventricular leukomalacia, and/or peri/intraventricular hemorrhage) and MBP less than 30 mm Hg at any time point in the first few postnatal days (3) and on more recent data aimed at maintenance of cerebral blood flow (CBF) measured by near-infrared spectroscopy (4, 5) Although the tenth centile for infants of all gestational ages by the third postnatal day is at or above 30 mm Hg, in more immature infants the 10th centile of MBP is lower than 30 mm Hg during the first 3 days (6). Another population based definition is a MBP less than the GA in weeks during the first postnatal days, which relates to the 10th centile for age in tables of normative data (6, 7). This statistical definition has been supported by guidelines for blood pressure management by several professional bodies (8). This definition is relevant mainly in the first 24–48 h of extrauterine life—after which time there is a gradual increase in the expected MBP such that most premature infants have a MBP >30 mm Hg and thus above their gestational age by postnatal day 3 (7). More recent cohorts of neonates have been published defining population based norms of blood pressure in the modern era of neonatal intensive care (9, 10).

## The Transitional Circulation in the Preterm Infant

The preterm infant frequently has a failure of complete closure of both fetal communications, probably due to immaturity of the structural mechanisms involved (11, 12). The persistence of fetal channels in the setting of decreasing pulmonary pressures leads to blood flowing preferentially from the aorta to pulmonary artery resulting in a relative loss of blood from the systemic circulation and overloading of the pulmonary circulation. The myocardium attempts to compensate by increasing the total cardiac output (13). A significant proportion of this increased blood flow passes through the ductus arteriosus (14), thus augmenting pulmonary blood flow. Early ductal constriction is variable, with some infants having a closed/small ductus arteriosus shortly after birth while others initially constrict, followed by an increase in size of the ductus and others demonstrate a persistent large ductus arteriosus with no evidence of any early constriction. In these infants with a wider open patent ductus arteriosus (PDA) and decreasing pulmonary pressures, there can be a rapid increase in ductal shunt, resulting in pulmonary blood flow more than twice the SBF in the first few postnatal hours (15). Excessive pulmonary blood flow can have clinical consequences, which include decreased systemic blood pressure and blood flow, increases in ventilatory requirements, or even pulmonary hemorrhagic edema and eventually pulmonary hemorrhage (15, 16).

Ventricular systolic function is defined by preload (filling of the ventricle with blood prior to contraction), contractility (intrinsic ability of myocardial fibers to contract), and afterload (combined resistance of the blood, the ventricular walls and the vascular beds). The preterm myocardium is less mature than the term infant with histological differences including less numbers of mitochondria and subsequently less energy stores (17). This results in limitation in the ability to respond to changes in the components of the cardiac output, in particular the afterload (18, 19). The myocardium of the preterm infant has a reduced ability to respond to postnatal period stress including increases in peripheral vascular resistance or afterload (20) with small changes having profound effects, particularly in the setting of inadequate preload or impaired contractility (18). Oxygen delivery is primarily related to the oxygen carrying capacity of the blood, oxygen content, and the volume of blood flow delivered to the organ, and this may be inadequate where there is cardiovascular impairment (21).

## Determinants of the Blood Pressure in the Preterm Infant

Arterial blood pressure is the product of cardiac output and the peripheral vascular resistance. The main influences on cardiac output are preload or blood volume and myocardial contractility. Peripheral vascular resistance is determined by vascular tone, which in the presence of an unconstricted ductus arteriosus includes not only the systemic peripheral vascular resistance but also pulmonary vascular resistance. Myocardial contractility is difficult to assess in the newborn as the accepted measures of contractility in the adult, such as the echocardiographic measure of fractional shortening, are adversely influenced by the asymmetry of the ventricles caused by the *in utero* right ventricular dominance. Newer techniques such as tissue Doppler imaging may overcome these limitations (20).

In the preterm neonate with a closed ductus arteriosus during the first 24–48 h, the relationship between MBP and cardiac output is poor (22). Many hypotensive preterm infants have a normal or high left ventricular output, thus compensating for reduced systemic blood pressure (22–24). One of the reasons for this apparent paradox is the presence of a hemodynamically significant ductus arteriosus, which causes an increase in left ventricular output while also causing a reduction in the overall systemic vascular resistance. Variations in the peripheral vascular resistance may cause a change in the underlying cardiac output that does not affect the blood pressure. This phenomenon makes it possible for two infants with the same blood pressure to have markedly different cardiac outputs.

### Gestational Age and Postnatal Age

All population-based collections of blood pressure data show that there is a major influence of gestational age on the blood pressure, with the most immature infants having the lowest MBP on the first day of life. Similarly, population based blood pressure nomograms show a significant influence of postnatal age on the blood pressure with a progressive increase in MBP each postnatal day (6, 25). This is probably related to changes in vascular tone mediated by humoral and sympathetic nervous system mediators, in addition to maturation of the fetal/neonatal myocardium such that it can respond better to afterload (19, 20).

### Blood Loss

Acute blood loss in the preterm infant is unusual and can result from prenatal events such as feto-maternal hemorrhage, antepartum hemorrhage, or twin–twin transfusion syndrome, intrapartum events such as a tight nuchal cord with differential vessel constriction resulting in an imbalance between blood flow to and from the fetus or postnatally from a large subgaleal hematoma or hemorrhage into an organ such as the liver or brain. Acute blood loss can result in significant hypotension but due to the immediate compensatory mechanisms of the cardiovascular system, these effects may be delayed and present only when the phase of decompensation occurs.

### Timing of Umbilical Cord Clamping

Both the volume of placental transfusion and the normal sequence of the transition can be affected by the timing of clamping of the umbilical cord with later clamping being associated with a smoother hemodynamic transition (in a preterm sheep model) (26). A delay in clamping the umbilical cord from 1 to 3 min is also associated with increased systemic blood pressure and a decreased need for cardiovascular support and blood transfusions as demonstrated in the systematic review of the effects of delayed cord clamping/placental transfusion (27). The largest RCT of delayed cord clamping to date (1,566 babies) has just been published (28) and a smaller subset of this trial had advanced cardiovascular monitoring and blood pressure measurements (29) which showed no increase in rates of treated hypotension in the early clamped arm.

### Positive Pressure Ventilation

Many preterm infants are exposed to positive pressure respiratory support in the first postnatal days, although there has been a progressive reduction in the use of mechanical ventilation, particularly as an early first line treatment. Positive end-expiratory pressure is often utilized to reduce the atelectasis resulting from collapse of unstable alveoli when surfactant is lacking, particularly in more immature infants. Function can be impaired by a reduction in the preload from reduced systemic or pulmonary venous return, and/or direct compression of cardiac chambers also resulting in a reduced stroke volume or an increase in afterload. In longitudinal clinical studies of blood pressure and blood flow, mean airway pressure has a consistently negative influence on both MBP and SBF (22, 30–32). More recent clinical studies have failed to demonstrate clinically relevant decreases in blood flow in the conventional range of pressure support (33–35).

### Patent Ductus Arteriosus

The classic signs of a PDA with increased pulse pressure and evidence of diastolic runoff have resulted in the perception that a PDA is mainly associated with a low diastolic blood pressure. However, several studies have suggested that the PDA can also be associated with both low diastolic and low systolic blood pressure. Thus, a PDA is one of the possible causes of systemic hypotension and PDA assessment should be part of the investigation of hypotension in the first days of life (36, 37). Clinical assessment of a PDA in the first postnatal days is difficult and cardiac ultrasound is often required for early diagnosis (38). The clinical signs of a PDA including systolic murmur, high volume pulses, and a hyperdynamic precordium only become easily detectable after day 3 of postnatal life (38). This increasing left to right shunt may also result in an increasing systemic to pulmonary shunt which can manifest as reduced blood pressure and/or SBF, even in the first few days of life.

## Assessment of Cardiovascular Compromise in the Preterm Infant

The assessment of cardiovascular adequacy and thus tissue oxygen delivery in the VLBW infant is more of a challenge than in infants and adults. Assessment often consists of a mainly clinical appraisal of the perfusion *via* capillary refill time (CRT) and urine output and the intermittent documentation of the pulse rate and blood pressure. The acid–base balance and evidence of lactic acidosis are further important adjuncts to this assessment but, unless serum lactate levels are serially monitored, monitoring changes in pH and base deficit may be misleading due to the increased bicarbonate losses through the immature kidneys. Additionally, these measures tend to reflect cardiovascular changes some hours previously rather than the current circumstances.

### Capillary Refill Time

Capillary refill time (CRT) is a widely utilized proxy of both cardiac output and peripheral resistance in adults and older children, as well as in neonates where normal values have been documented (39). A study investigating the relationship between a measure of SBF and CRT in VLBW infants showed that a CRT of ≥3 s had only 55% sensitivity and 81% specificity for predicting low SBF. However, a markedly increased CRT of 4 s or more was more closely correlated with low blood flow states (40).

### Urine Output

Urine output is useful in the assessment of cardiovascular well-being in the adult; however, the immature renal tubule in VLBW infants is inefficient at concentrating the urine and therefore has an impaired capacity to appropriately adjust urine osmolality and flow in the face of high serum osmolality (41). Even if the glomerular filtration rate is decreased markedly, there can be little change in urine output. Accurate measurement of urine output is not easy in VLBW infants, requiring collection *via* a urinary catheter or *via* a collection bag, both techniques being invasive.

### Pulse Rate

Preterm infants generally have a faster baseline heart rate and immature myocardium and autonomic nervous system such that the cardiovascular response to hypovolemia is different to that in older children and adults. A rising pulse rate is usually indicative of hypovolemia in the adult. The mechanism relies on a mature autonomic nervous system, with detection of reduced blood volume and then blood pressure *via* baroreceptors and a subsequent increase in the heart rate in an attempt to sustain appropriate cardiac output. Heart rate is affected by many things in the immediate postnatal period so it cannot be relied upon as an accurate assessment of cardiovascular status.

### Blood Pressure

Arterial blood pressure can be measured invasively using either an umbilical artery catheter placed in the descending aorta or an arterial catheter placed in a peripheral artery such as the radial or posterior tibial artery. There is good correlation between blood pressure obtained *via* a peripheral artery catheter versus that obtained *via* the umbilical artery (42). The agreement between direct and indirect (noninvasive) measures of blood pressure is generally also good (43–48). However, the noninvasive technique is more problematic in the preterm infant as it is more dependent on choice of the appropriate cuff size and is non-continuous (49, 50). With the increased use of non-invasive respiratory support even in the smallest infants, access to invasive blood pressure is becoming less common resulting in the need for other ways to assess perfusion and cardiovascular adequacy. There is an increasing tendency to utilize non-invasive BP measurement in our unit as the perceived risks of arterial lines are balanced with the decreasing incidence of hypotension in even the smallest babies.

### Metabolic Acidosis/Lactic Acidosis

When oxygen delivery to organs is less than organ oxygen requirements and the compensatory mechanisms are fully utilized, there is a change to anaerobic metabolism at the cellular level. As adequate cardiovascular function is a key part of oxygen delivery, reduced SBF may result in increasing serum lactate levels. A combined lactate value of more than 4 mmol with prolonged CRT more than 4 s predicts low SBF with 97% specificity (51). There are associations between raised serum lactate levels and illness severity and mortality in ventilated neonates, specifically those with respiratory distress syndrome (52–59). The normal lactate level is <2.5 mmoL/L, and as the serum lactate level increases above this threshold, there is an increase in mortality (57, 58).

### Cardiac Output and SBF

The size of neonates, particularly those less than 30 weeks gestation, with the associated difficulty of placing intracardiac catheters, has meant that the use of more invasive hemodynamic measures such as pulmonary artery thermodilution and mixed venous oxygen saturation monitoring has not been possible. Another issue specific to premature infants is the potential inaccuracy of the dye dilution and thermodilution method in the presence of intracardiac shunts through the ductus arteriosus and the foramen ovale. Noninvasive methods of measuring cardiac output, particularly using Doppler ultrasound, have become more popular, aided by improvements in picture resolution and reductions in ultrasound transducer size. Newer approaches such as impedance electrical cardiometry, which allows continuous beat-to-beat assessment of cardiac output are now becoming available and may facilitate the continuous collection of cardiac output for real-time decision making (60).

Normal MBP does not mean normal LV output or cerebral blood flow in preterm infants, including in those who have a closed ductus arteriosus (22, 24, 61). This observation has led to the development of bedside cardiac ultrasound techniques directed at measuring cardiac output/SBF and assessing the function of the heart as well as allowing assessment of the size of fetal shunts (62). Assessment of SBF in the preterm infant is problematic due to the persistence of the fetal shunts (PFO and PDA). Increased blood flow through the PDA (left to right ductal shunt) results in an increased LV output as the output is measured at the aortic valve, prior to the PDA connection to the systemic circulation. Similarly, left to right shunt through a patent foramen ovale results in an increased RV output as the output is measured at the pulmonary valve, after the point where the PFO shunt combines with the systemic venous return (15). Thus, neither LV nor RV output are truly representative of SBF. To overcome this limitation, we developed the measure of superior vena cava (SVC) flow which assesses cardiac input and is unaffected by the fetal shunts (63).

Low SBF, as measured *via* SVC flow or return of blood from the upper body to heart (63), is seen in up to 35% of VLBW infants in the first 24 h. Not all of these infants will have hypotension initially, though many (up to 80%) will develop it (63). More recent studies of the incidence of low SVC flow show a reduced incidence (18–21% of ELBW infants) possibly reflecting changes in delivery room care, respiratory management, and perhaps fluid management of the neonate (29, 64, 65). SBF may fall dramatically in extremely premature infants during the first hours after delivery and this reduction in flow is usually associated with an increase in peripheral vascular resistance/afterload (19). A substantial proportion of these infants will initially have a “normal” blood pressure (i.e., they are in “compensated shock”). Hypotension may also be associated with normal or even a high SBF as frequently occurs in the preterm infant with persisting hypotension after the first postnatal days or those with “hyperdynamic” sepsis (66). These infants generally have low systemic vascular resistance with peripheral vasodilation.

## Conclusion

Appropriate assessment and treatment of the preterm infant with cardiovascular impairment or shock requires the clinician to obtain adequate information about the etiology and underlying physiologic determinants of the condition. Ultrasound evaluation can provide information about the size and shunt direction of the ductus arteriosus, the function of the myocardium and its filling as well as a cross sectional measurement of the cardiac output. This information is valuable in understanding underlying physiology and deciding on management strategies for newborn infants with cardiovascular instability and/or impairment.

## Author Contributions

This article was conceived and written by MK.

## Conflict of Interest Statement

The authors declare that the research was conducted in the absence of any commercial or financial relationships that could be construed as a potential conflict of interest.
